# Repair of total penile skin loss after ritual circumcision using a scrotal flap: a case report

**DOI:** 10.11604/pamj.2022.42.152.35813

**Published:** 2022-06-24

**Authors:** Sultan Qaid, Faisal Ahmed, Ebrahim Al-shami, Qasem Alyhari, Saif Ghabisha, Mohammad Reza Askarpour, Zakaria Al-mekhlafy, Essam Hadwan

**Affiliations:** 1 Urology Research Center, Al-Thora General Hospital, Department of Urology, School of Medicine, Ibb University of Medical Science, Ibb, Yemen,; 2 Department of General Surgery, School of Medicine, Ibb University of Medical Science, Ibb, Yemen,; 3 Department of Urology, School of Medicine, Shiraz University of Medical Sciences, Shiraz, Iran,; 4 Student Research Committee, School of Medicine, Ibb University of Medical Sciences, Ibb, Yemen

**Keywords:** Skin loss, ritual circumcision, scrotal flap, case report

## Abstract

Ritual circumcision is associated with a high rate of complications, mainly if performed by an untrained practitioner. Furthermore, excessive skin removal is a rare complication of this procedure that results in penis “trapping” underneath the skin and future sexual dysfunction. Here, we presented a 45-day-old Yemeni newborn with a trapped penis due to total loss of penile skin during a ritual circumcision performed by a traditional untrained practitioner using the guillotine technique one month ago. The patient underwent surgical exploration, and the penis was deliberated, released, and the skin defect was repaired with a single-step scrotal flap advancement over the penile shaft. At the six-month follow-up, the outcome was both functional and cosmetically satisfying. In conclusion, we recommend that the circumcision procedure be performed at the very least by an educated and skilled health professional. Additionally, a scrotal advancement flap is still an option in significant penile skin loss cases.

## Introduction

Circumcision is one of the oldest and the most common surgical procedures performed regularly on most newborn infants in Muslim and Jewish countries. It is performed for various religious, cultural, and medical reasons [1]. The complication rate of this procedure is between 0.2% and 5%. It can range from mild loss of penile skin to significant complications such as glans amputation, total penile loss, and even death [2]. Complication rates depend on different elements, such as comorbid factors, type of anesthesia, practitioner's expertise, and child age [2, 3]. Since few cases of total penis skin loss in circumcision procedures have been reported, records of these complications and its consequences are required [4, 5]. We present a 45-day-old infant with complete penile skin loss following ritual circumcision, which was successfully treated with a scrotal flap.

## Patient and observation

**Patient information:** a 45-day-old Yemeni newborn was referred to our facility with a trapped penis due to total penile skin loss after a ritual circumcision by a traditional untrained practitioner using the guillotine technique performed one month ago.

**Figure 1:**
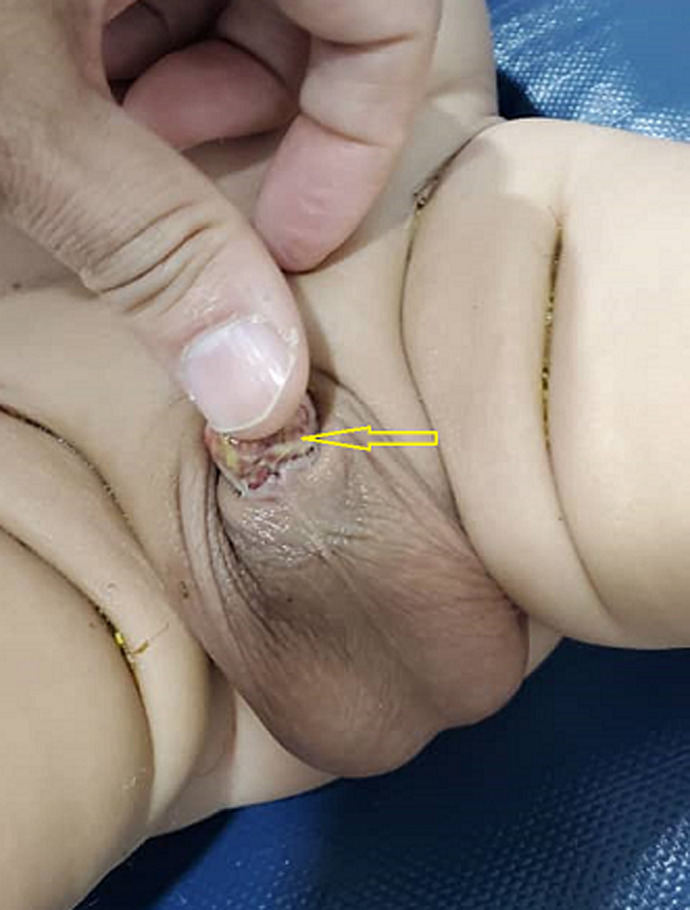
preoperative photo showing trapped penis

**Clinical findings:** examination of the external genitalia showed a trapped penis (the shaft of the penis is abnormally secure to the surrounding scrotal skin) (Figure 1). The rest of the examination was uneventful, and the final diagnosis was complete penile skin loss.

**Diagnostic assessment:** laboratory tests, including complete blood counts, liver function tests, coagulation tests, and renal function tests, were within normal limits. The abdominal ultrasonography was normal.

**Figure 2:**
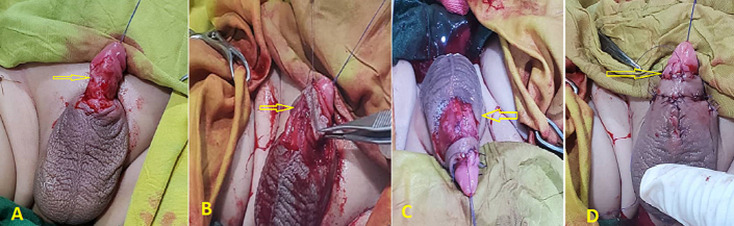
A) total penile skin loss after release (arrow); B) dorsal view of the penile shaft with total skin loss; C) proximal view of the penile shaft was covered by a scrotal flap (arrow); D) dorsal view of the penile shaft was covered by a scrotal flap (arrow)

**Figure 3:**
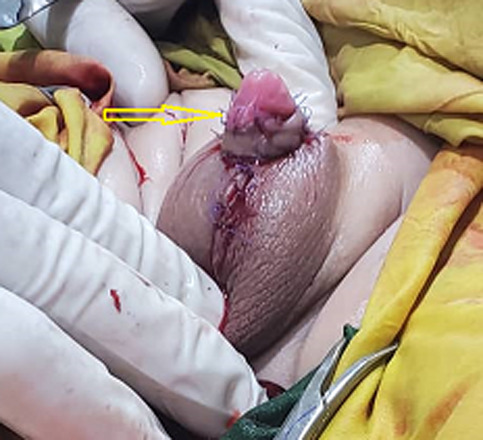
postoperative appearance of the penis after the surgery

**Surgical procedure:** the patient underwent reconstructive surgery under general anesthesia. Firstly, the penis was deliberated and released from surrounding tissue. Then, the skin on the median scrotal raphe was incised, followed by subcutaneous dissection of the scrotal skin on both sides of the median raphe while keeping the vascular pedicles. Then the scrotal flap was gently moved over the penis and wrapped around the penile shaft (Figure 2). Finally, the skin was sutured using an absorbable vicryle 4/0 suture (Figure 3).

**Figure 4:**
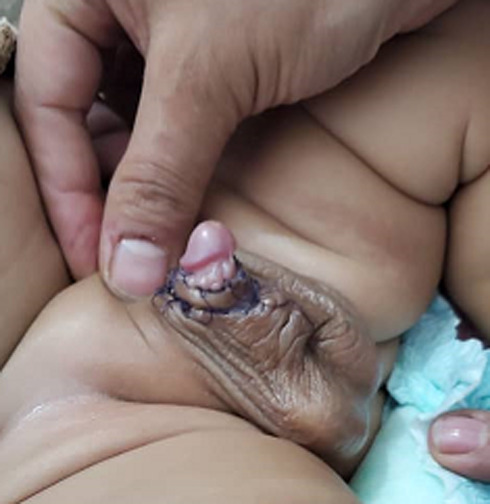
postoperative appearance of the penis one month after the surgery

**Follow-up and outcome:** the patient received ceftriaxone 100 mg/kg for five days. The postoperative period was uneventful, and he was discharged on the second postoperative day. At the six-month follow-up, the outcome was both functional and cosmetically satisfying. (Figure 4).

**Patient perspective:** after six months of treatment, the patient family was satisfied with the result of the treatment and postoperative patient care.

**Informed consent:** written informed consent was obtained from the patient family for participation in our study.

## Discussion

Circumcision is among the most common surgical procedures performed on newborn infants in Muslim and Jewish countries. Families primarily seek this procedure based on religious, cultural, and medical purposes [1]. Complications of circumcision vary depending on several elements such as comorbid factors, type of anesthesia, practitioners' training and experience, and child age [2, 3]. While, in developed countries, most complications are related to the type of anesthesia, in developing countries, most complications are caused by untrained practitioners, as in our case [1, 3, 6].

Denudation of the penile shaft as a circumcision complication is pretty infrequent, with reported rates ranging from 1% to 3% [7]. Several mechanisms are proposed as an explanation for this rare complication. It can occur due to direct excessive skin excision by untrained individuals, such as in our case, or postoperative skin infections and severe debridement or skin necrosis [8]. Numerous reconstruction techniques are suggested based on the severity of skin loss, varying from directed healing to skin grafts using thin or thick flaps or fasciocutaneous advancement flap, utilizing a flap from the inguinal, femoral, or even scrotal region [4].

One of the essential factors for graft survival is a well-vascularized “bed,” adequate tension, and solid dressing compression. A skin graft could be obtained from non-hirsute skin with sufficient elasticity to allow the penis to work effectively in flaccid and erect states and have an excellent tactile sensation. The scrotal graft is the most similar, but the skin is hairy and unsuitable for excessive skin loss. The split-thickness skin graft has been used successfully in numerous reports, but the graft is susceptible to shrinking and infection [4, 8]. The full-thickness skin graft is a successful reconstructive method with improved skin elasticity. Because hypodermal tissue is kept in place in elective cases with a “trapped” penis, the graft survival is significantly higher than those with a traumatic degloved penis, where the skin with hypodermal tissue is removed, and Buck's fascia is exposed [3, 4, 9].

In our case, we chose a skin advancement flap using the scrotal flap while keeping the tissues' vascularization intact; Gao *et al*. and Nwaha *et al*. reported similar methods [8, 9]. Postoperative care, such as frequent dressings with normal saline and avoiding erections, aimed to ensure appropriate flap healing and avoid infections. Cosmetic appearance and sexual function are important factors in long-term follow-up [8]. In our case, the patient family was satisfied regarding cosmetic factors. As mentioned in previous studies, ritual circumcision by untrained practitioners acts as a primary cause of higher complication rates in our society. The government can play a huge role in resolving this problem by providing training programs or limiting the untrained practitioners from performing circumcision, which remains an attractive option for low-income families due to cheaper costs [1, 6]. The prevention will begin with public education and training for all those involved in circumcision, including traditional practitioners, paramedics, medical doctors, and surgeons [1, 3].

## Conclusion

We recommend that performing circumcision be limited to educated and skilled health professionals. Additionally, in significant penile skin loss cases, we suggest a scrotal advancement flap as a valid reconstructive option with benefits such as an aesthetic scar, skin with better functionality, and shorter hospitalization.
